# Lessening of porcine epidemic diarrhoea virus susceptibility in piglets after editing of the CMP-N-glycolylneuraminic acid hydroxylase gene with CRISPR/Cas9 to nullify N-glycolylneuraminic acid expression

**DOI:** 10.1371/journal.pone.0217236

**Published:** 2019-05-29

**Authors:** Ching-Fu Tu, Chin-kai Chuang, Kai-Hsuan Hsiao, Chien-Hong Chen, Chi-Min Chen, Su-Hei Peng, Yu-Hsiu Su, Ming-Tang Chiou, Chon-Ho Yen, Shao-Wen Hung, Tien-Shuh Yang, Chuan-Mu Chen

**Affiliations:** 1 Division of Animal Technology, Animal Technology Laboratories, Agricultural Technology Research Institute, Xiangshan Dist., Hsinchu, Taiwan, R.O.C; 2 Department of Life Sciences, National Chung Hsing University, South Dist., Taichung, Taiwan, R.O.C; 3 Division of Animal Medicine, Animal Technology Laboratories, Agricultural Technology Research Institute, Xiangshan Dist., Hsinchu, Taiwan, R.O.C; 4 Department of Veterinary Medicine, College of Veterinary Medicine, National of Science and Technology, Pingtung, Taiwan, ROC; 5 Division of Animal Industry, Animal Technology Laboratories, Agricultural Technology Research Institute, Xiangshan Dist., Hsinchu, Taiwan, R.O.C; 6 Department of Biotechnology and Animal Science, National Ilan University, Yilan, Yilan, Taiwan, R.O.C; 7 The iEGG and Animal Biotechnology Center, National Chung Hsinh University, Taichung, Taiwan, R.O.C; University of Connecticut, UNITED STATES

## Abstract

The porcine epidemic diarrhoea virus (PEDV) devastates the health of piglets but may not infect piglets whose CMP-N-glycolylneuraminic acid hydroxylase (CMAH) gene is mutated (knockouts, KO) by using CRISPR/Cas9 gene editing techniques. This hypothesis was tested by using KO piglets that were challenged with PEDV. Two single-guide RNAs targeting the CMAH gene and Cas9 mRNA were microinjected into the cytoplasm of newly fertilized eggs. Four live founders generated and proven to be biallelic KO, lacking detectable N-glycolylneuraminic acid (NGNA). The founders were bred, and homozygous offspring were obtained. Two-day-old (in exps. I, n = 6, and III, n = 15) and 3-day-old (in exp. II, n = 9) KO and wild-type (WT, same ages in respective exps.) piglets were inoculated with TCID_50_ 1x10^3^ PEDV and then fed 20 mL of infant formula (in exps. I and II) or sow’s colostrum (in exp. III) every 4 hours. In exp. III, the colostrum was offered 6 times and was then replaced with Ringer/5% glucose solution. At 72 hours post-PEDV inoculation (hpi), the animals either deceased or euthanized were necropsied and intestines were sampled. In all 3 experiments, the piglets showed apparent outward clinical manifestations suggesting that infection occurred despite the CMAH KO. In exp. I, all 6 WT piglets and only 1 of 6 KO piglets died at 72 hpi. Histopathology and immunofluorescence staining showed that the villus epithelial cells of WT piglets were severely exfoliated, but only moderate exfoliation and enterocyte vacuolization was observed in KO piglets. In exp. II, delayed clinical symptoms appeared, yet the immunofluorescence staining/histopathologic inspection (I/H) scores of the two groups differed little. In exp. III, the animals exhibited clinical and pathological signs after inoculation similar to those in exp. II. These results suggest that porcine CMAH KO with nullified NGNA expression are not immune to PEDV but that this KO may lessen the severity of the infection and delay its occurrence.

## Introduction

Porcine epidemic diarrhoea (PED) was first recognized as an enteric disease in 1971 by the British veterinarian Oldham [1]; subsequently, the PED virus (PEDV) was isolated by Pensaert and de Bouck [2] at Ghent University in Belgium. Since then, PEDV-associated diarrhoea has been widely detected in Europe. In Asia, it was reported in 1982 [3], and it has subsequently greatly impacted the Asian pork industry. In China during 2010 and 2011, over one million nursing piglets were lost due to PEDV-associated diarrhoea [4]; in 2013, PEDV emerged in Korea and the USA [5–7] as well as in Taiwan [8], causing great economic losses and continuing to spread as an epidemic.

PEDV and transmissible gastroenteritis virus (TGEV) are members of the *Coronaviridae* family and the alpha coronavirus group. The PEDV genome consists of a positive single-stranded RNA approximately 28 kb in length that contains 7 open reading frames (ORF), including ORF1a, ORF1b, and ORF2-6 [9]. The viral particles are coated with S-protein, a type I membrane protein. The protein forms spikes on the viral surface that are used to infect host cells and also bears highly antigenic domains and could theoretically be used to develop a high-titre neutralizing PEDV vaccine [6, 10]. However, Sun et al. [11] found that the sequence of this region is highly variable, a characteristic that is likely to reduce the efficiency of conventional commercial vaccines. Furthermore, the S-protein is a glycoprotein that undergoes complicated post-translational modifications that result in antigen diversity and create obstacles to the development of a PEDV vaccine [10].

The pathway of PEDV infection occurs mainly through the S-protein. PEDV first contacts sialic acids (neuraminic acid, NA) in host intestine [12] and then infects the villi by binding to aminopeptidase N (APN) on epithelial cells [13, 14]. These findings suggest that NA is the first glycoprotein receptor and that APN is the second one for PEDV during infection of the host intestine [15]. A similar process occurs during infection by TGEV [12]; on the other hand, porcine respiratory coronavirus (PRCV) loses its ability to infect the host intestine due to mutation and deletion of the S-protein genomic region occur [16]. Since viral genomic sequences of S-protein are generally variable and unstable, but in mammals, e.g., pigs, the codon sequences of their receptor are more stable and allow to be manipulated specifically by gene editing (GE). As mentioned above, PEDV infects the host via NA and APN, and NA has been shown to play an important role in host immune function and infection by pathogens [17, 18]. Human cells synthesize N-acetyl NA (NANA) but not N-glycolyl NA (NGNA), due to CMP-N-glycolylneuraminic acid hydroxylase (CMAH) insert an oxygen into the acetyl group of NANA converted to the glycolyl of NGNA [19] and the human CMAH gene has mutated during 2.5–3.0 million years of evolution [17]. We suggest that, analogous to the way in which human evolution has eliminated the NGNA receptor for PEDV, the CMAH gene of domestic pigs might be artificially mutated by gene editing technology to produce resistance to PEDV infection. The APN gene is not proposed as a target because it is essential for dipeptide digestion and amino acid absorption.

Currently available technologies for gene editing (GE) include the use of ZFN (zinc finger nuclease) [20], TALEN (transcription activator-like effector nuclease) [21], and CRISPR (clustered regularly interspaced short palindromic repeat)/Cas9 (CRISPR-associated (Cas) endoribonuclease 9) [22]. Due to the availability of convenient techniques for constructing and editing vectors and the fact that Cas9 is a universal enzyme that can be constructed separately to guide/target vectors, use of the CRISPR/Cas9 system for GE is currently more popular than use of the ZFN and TALEN systems. Furthermore, GE can be simultaneously conducted on multiple sites or genes with the same Cas9 to achieve different targeting purposes or reduce the risks of off-targeting [23–25]. We have established TALEN [26] and CRISPR/Cas9 [27, 28] systems for direct microinjection of GE vectors to generate α1, 3-galactosyltransferase (GGTA1) mutant pigs. In this study, direct microinjection of two single-guide RNA and Cas9 mRNA vectors into the cytoplasm of pronuclear porcine embryos was used to generate CMAH mutant pigs with null expression of NGNA, and the possibility of obtaining mutant piglets that are resistant to infection by PEDV was examined.

## Materials and methods

### Animals and animal care

Landrace mature gilts at least 120 to 150 kg in weight or sows and their neonatal piglets were used in this study. All animals were reared in a station free from specific pathogens (atrophic rhinitis, *Mycoplasma hyopneumoniae*, pseudorabies, *Actinobacillus pleuropneumoniae*, swine dysentery, scabies, classical swine fever, foot and mouth disease and porcine reproductive and respiratory syndrome). The gilts or sows were housed indoors on concrete floors, and the accommodation was artificially lit (450–600 lux for 9 hours a day) and exposed to window sun light. The animals were fed a restricted (4% body weight) commercial diet formulated to meet the requirements recommended by the National Research Council [29] and had *ad libitum* access to water.

All animals were managed and treated with permission from the Agricultural Technology Research Institute (ATRI) (IACUC104004). The use of the animals and the PEDV challenge protocol, including the specific criteria used to determine when piglets should be euthanized, were approved by the confirmations of IACUC committee of ATRI (IACUC105063) and of National Pingtung University of Science and Technology (NPUST) (NPUST-105-060). All the personal involved in the study have own a certificate from the experimental animal course in pig care or handling. Three *in vivo* PEDV-challenged experiments were conducted and in total of 60 piglets were used in this study. During the 3 days experiment, all animals’ health and behavior were monitored by the research staffs every 4 h and veterinarian once per day. Part of the piglets (1 KO and 3 WT in exp. I; 9 KO and 7 WT in exp. II; and 1 KO before and 1 KO in exp. III) died before meeting criteria for euthanasia (during 72 hpi of PEDV). During the *in vivo* experiments, welfare considerations also were taken, including efforts to minimize suffering and distress and use of analgesics (3 mg/kg ketoprofen, intramuscular injection, once per day) if need. When piglets exhibited abnormal clinical behavior such as falling down and pumping, labored breathing, or sudden lethargy were observed, the animals shall be seen reached humane endpoint criteria and immediately euthanized by intramuscular injection of 5 mg/kg Zoletil (Virbac, France) and exsanguination.

### Treatment of donors and recipients

Six donors were synchronized and induced to super-ovulate by being fed a ration supplemented with Regumate (containing 0.4% Altrenogest; Intervet, MSD, France) for 15 days to synchronize their oestrus cycles and then being intramuscularly injected with PMSG (1,750 IU) and hCG (1,500 IU), 78 h apart, to induce oocyte maturation and ovulation. After hCG injection, the animals were artificially inseminated and sacrificed 30 to 36 h or 54 to 56 h later, and fertilized eggs were harvested from their oviducts. Three recipients were synchronized and ovulation-induced by the same methods as donors except that all treatments were delay 12 h and the dosage of PMSG and hCG was reduced to 1,500 IU and 1,250 IU, respectively, and insemination did not occur. When the fertilized eggs arrived at a nearby laboratory, CRISPR/Cas9 RNA was microinjected into the cytoplasm; the eggs were then surgically transferred to the oviduct of a recipient from the end of the infundibulum by exposure of the uterine horn and oviducts within 3 to 4 h. The recipients were raised normally but treated with special care, particularly during farrowing.

### Pig embryo manipulation and microinjection

The recovered newly fertilized eggs were centrifuged at 15,000xg for 10 to 15 min at 25°C to expose their pronuclei. The pronuclear embryos were added to a 20 μL microdroplet of D-PBS in a glass slide chamber and covered with mineral oil. The micro-manipulation was conducted under an inverted DIC (differential interference contrast) microscope at 200 to 300 x magnification. Each embryo was held in the proper position to reveal the pronucleus, and a mixture of single-guide RNA directed against two sites (sgRNA, 10 ng/μL each) and Cas9 RNA (70 ng/μL) was microinjected into the cytoplasm near the pronucleus using a capillary needle with steady flow.

### Animals breeding

The confirmed CMAH KO pigs (founders) were raised by following the husbandry practices for breeding stocks of SPF herd. When reached puberty and showed their second estrous cycle, the females were thereafter estrus synchronized by Regumate feeding and withholding. The female founders were then artificially inseminated with fresh extended semen collected from the male littermate founder to generate homozygous offspring for the study.

### Construction of CMAH gene-specific sgRNA knockout and Cas9 vectors

**Fig 1:**
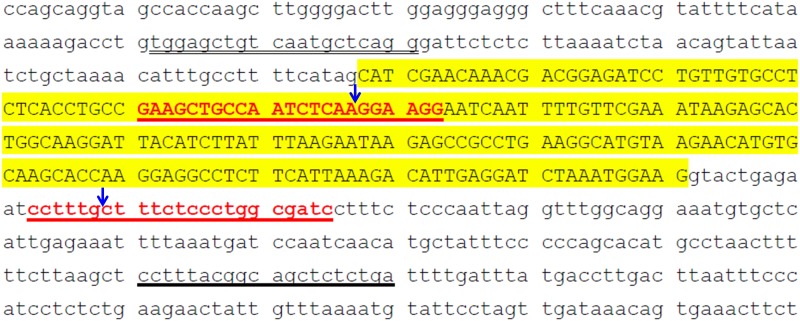
Construction of CMAH gene-edited vectors. The porcine CMAH gene editing sites were designated on exon 2 (sense strand, red capital letters underlined in red) and intron 2 (antisense strand, red letters underlined in red). The sequences underlined in black are PCR primers (CMAH Ex2 F and CMAH Ex2 R). The sequences shown in large capital letters with yellow shading are exon 2. The blue arrows indicate the gene editing sites.

The codon region of the porcine CMAH gene includes 14 exons; exon 1 contains 8 bp, and exon 2, which is 204 bp in length, is the largest exon (Fig 1, large capital letters shaded in yellow). After verifying the sequences of exon 2 and introns 1 and 2 of the CMAH gene, we chose two GN_19_NGG Cas9 specific sequences; one of these has a sense strand site on exon 2, and the other has an antisense strand site located on intron 2 (Fig 1, characters underlined in red). According to the sequences of the selected sites, two synthetic DNA primer pairs (shown in Table 1) were annealed as double-stranded DNA fragments, digested with BsalI and cloned into the ppU6-(BsaI)_2_-sgRNA vector [30]; in this way, two sgRNAs, ppU6-(CMAH ex2)-sgRNA and ppU6-(CMAH in2)-sgRNA, were constructed. Cas9 in the pCX-Flag_2_-NLS1-Cas9-NL-S2 vector was constructed by Su et al. [30]. To make it possible to use the RNAs for gene editing, pT7-Flag_2_-NLS1-Cas9-NLS2-3’pA, pSP6-(CMAH ex2)-sgRNA and pSP6-(CMAH in2)-sgRNA were constructed for *in vitro* transcription (S1 Fig). To prepare capped and poly-A tailed Cas9 mRNA, *Hin*dIII linearized pT7-Flag_2_-NLS1-Cas9-NL-S2-3’pA DNA template was transcribed by mRESSAGE mMACHINE T7 Transcription Kit (Ambion, AM1344, Carlsbad, CA, USA). To prepare CMAH single-guide RNAs, both of the *Bgl*II linearized pSP6-(CMAH ex2)-sgRNA and pSP6-(CMAH in2)-sgRNA DNA templates were transcribed by MEGAscript SP6 Kit (Ambion, AM1330, Carlsbad, CA, USA). All of the transcribed RNA products were further purified by the MEGAclear Transcription Clean-Up Kit (Ambion, AM1908, Carlsbad, CA, USA) for microinjection.

**Table 1:** Primer pairs used to construct sgRNA expression vectors.

Primer	Sequence
pCMAH exon 2F	CGTC GAAGCTGCCAATCTCAAGGA GTTTTAGAGCTAGAAAT
pCMAH exon 2R	TGCTATTTCTAGCTCTAAAAC TCCTTGAGATTGGCAGCTTC
pCMAH intron 2F	CGTC GATCGCCAGGGAGAAAGCAA GTTTTAGAGCTAGAAAT
pCMAH intron 2R	TGCTATTTCTAGCTCTAAAAC TTGCTTTCTCCCTGGCGATC

### Screening of CMAH gene mutant pigs

Genomic DNA of all pigs delivered from foster dams or founders was isolated from tissue obtained from the piglet’s tails and purified using a genomic DNA purification kit (Fermentas/Thermo). The CMAH mutant pigs were first screened by PCR using 0.1 μg of genomic DNA and 0.25 μM each of CMAH Ex2 F (TGGAGCTGTCAATGCTCAGG) and CMAH Ex2 R (TCAGAGAGCTGCCGTAAAGG) primers (Fig 1) annealed at 55°C. Wild-type or site-mutated pigs produced a ~439-bp amplicon, and biallelic simultaneously mutated animals displaying the 161-bp deletion produced a ~278-bp amplicon. For further confirmation, all PCR products were verified by PCR product-direct sequencing (PDS) and PCR product/TA cloning/sequencing (PTS); from the latter, at least 6 colonies were picked and sequenced. DNA primer synthesis and DNA sequencing were conducted by Mission Biotech Ltd. (Taipei, Taiwan). The sequencing data were analysed using BioEdit software.

### Analysis of NGNA/NANA by HPLC

Samples of ear, tail and small intestine weighing approximately 100 mg were cut into small pieces in MQ water and incubated at 95°C for 30 min. After the samples had cooled to room temperature, 0.5 M H_2_SO_4_ was added to a final concentration of 25 mM. The mixtures were incubated at 80°C for 1 h to release the sialic acids from the samples. After centrifugation, the supernatant was collected, an equal volume of DMB (1,2-diamino-4,5-methylenedioxybenzene, Sigma-Aldrich, Inc.) solution (1.6 mg DMB in 1 mL of 1.4 M acetic acid, 0.75 M 2-mecaptoethanol and 18 mM sodium hydrosulfite solution) was added, and the mixture was incubated at 80°C for 2 h to label the sialic acids. The labelled NGNA and NANA used as standards were prepared as 1 mg/mL solutions and reacted under the same labelling conditions. The DMB-labelled sample was injected onto a Waters^™^ HPLC system (Waters 2475 Multi-wavelength Fluorescence Detector, Waters 717 plus Autosampler and Waters 600 Controller) with the Discovery BIO wide Pore C18 (5 μm, 4.6 x 25 cm) column. The analysis was performed using an isocratic mobile phase of methanol:acetonitrile:H_2_O (7:9:84) at a flow rate of 0.6 mL/min; the fluorescence detector was set at an excitation wavelength of 373 nm and an emission wavelength of 448 nm. (dx.doi.org/10.17504/protocols.io.zd6f29e).

### PEDV challenge

#### Piglet treatment and facility

All CMAH KO neonatal piglets (refer Results) were delivered from three F0 female founders that were served by the male F0 founder; thus, all founders were half or full sibs. All founders were biallelic CMAH mutants carrying a biallelic 161-bp deletion (D/D type) or one allele deleted and the other mutated (D/M type) genetic background. The D/D type and/or D/M type piglets were used as described in the experimental section. The control piglets were non-gene-edited piglets that were concurrently delivered from wild-type sows at the same farm.

PEDV challenge was conducted in a negatively air-conditioned animal facility at the NPUST. The pens were equipped with stainless mesh floors that allowed the faeces to drop down to a collection plate. The room temperature was set at 30°C, and each pen was equipped with two extra electric power bubs.

During 4 h shipping (from farm to challenge facility), the piglets were kept at 25°C in dark containers. When they arrived at the challenge room, the mutant and wild-type piglets were grouped and placed in different pens. Approximately one hour later, all piglets were oral inoculated with PEDV, which diluted in commercial baby formula that had been reconstituted with warm drinking water. In experiments I and II, the animals in each pen had free access to 200 mL of fresh prepared baby formula and clean tap water that was changed every 4 h. In exp. III, PEDV was diluted with KO or wild-type sow’s milk obtained 2 days after parturition, and no milk was offered; instead, fresh drinking water was offered and changed every 4 hours. Other treatments were as described in experimental design III.

#### Experimental design

**Exp. I: Challenge of 2-day-old neonatal piglets with nv-PEDV**. In total, 6 D/D type and 6 wild-type piglets were used for PEDV challenge, and one D/D type and one wild-type piglet without virus treatment were used as controls; the latter were not housed with the infected piglets. All neonatal piglets were nursed for approximately 20 h to allow intake of colostrum and then delivered to a negatively air-conditioned facility.

**Exp. II: Challenge of 3-day-old neonatal piglets with nv-PEDV**. In this trial, 8 D/D and 1 D/M type mutant piglets and 9 wild-type piglets were used for PEDV challenge, and one D/D mutant piglet and one wild-type piglet without virus treatment served as controls. All of the neonatal piglets were nursed for approximately 44 h to permit intake of colostrum and dam’s milk. The detailed conditions of the PEDV challenge were the same as those used in experiment I.

**Exp. III: Challenge of 2-day-old neonatal piglets with nv-PEDV followed by extended feeding of sows’ colostrum**. In this trial, 8 D/D and 3 D/M type mutant piglets and 12 wild-type piglets were used for PEDV challenge, and one D/D type piglet and one wild-type piglet without virus treatment served as controls. All neonatal piglets were nursed for approximately 20 h to permit intake of colostrum and then delivered to a negatively air-conditioned facility. In this trial, the piglets were orally inoculated with PEDV as in experiment I and II and were not fed commercial baby cow milk; instead, they were fed their dams’ or other founder’s milk that had been collected within 20 h. From 4 to 24 h post PEDV inoculation (hpi), the piglets were fed 20 mL of sow’s milk by hand every 4 h; whole milk was fed at 4 and 8 hpi, and skim milk was fed from 12 to 24 hpi. From 24 hpi to 72 hpi, 20 mL of lactated Ringer’s solution supplemented with 5% glucose was fed to each piglet every 4 h. The piglets were randomly allocated to sacrifice at 24 hpi (3 piglets), 48 hpi (3 piglets), or 72 hpi (6 piglets), and the small intestines were sampled.

### Preparation of new variant-PEDV for use in PEDV challenge

The new variant-PEDV (nv-PEDV) was isolated from a field case that occurred at Jimei farm in Yunlin County in central Taiwan in February 2015. Almost all of the affected one-week-old piglets died of watery diarrhoea. The aetiology of the disease was confirmed to be a virulent strain of PEDV (it was thereafter designated the Jimei strain); the sequence of this strain is almost identical to that of the strain that caused the epidemic outbreak of PEDV in the US in 2014 [31]. Although nv-PEDV can replicate in the Vero cell line, the nv-PEDV used in the challenge was prepared by oral inoculation of new born piglets that had not received colostrum to maintain its pathogenicity. The piglets were raised in a warm isolated chamber and were hand-fed fresh milk every six hours. Diarrhoea began to occur at 16–20 h after viral inoculation. The piglets were sacrificed 16–24 h after the observation of diarrhoea symptoms. The small intestinal content was collected by injection of 50 mL of DMEM supplemented with 10x P/S into the lumen followed by massage and extrusion from one end to the other end. The intestinal content was filtered through stainless mesh to clarify the content. Finally, the sample was centrifuged at 3000xg to precipitate all cellular debris, and the supernatant was collected and divided into 5-mL portions in sterile conical tubes. Three small fragments of intestine were subjected to paraffin-embedded tissue sectioning and IHC to confirm the presence of PEDV in intestinal epithelial cells (TGEV and rotavirus detection was also performed, and both tests were negative). A TCID_50_ was used according to standard virological methods to determine the viral content of the Jimei PEDV virus preparation used in the challenge study [32]. The virus was maintained at -80°C until the challenge study was performed. Inoculation of the animals with PEDV was conducted as described by Jung et al. [7]. In brief, 10^3^ TCID_50_/mL of frozen nv-PEDV stock was thawed at hand temperature, and 10 mL of the thawed stock was mixed with 90 mL of reconstituted commercial baby milk or sow’s milk by repeatedly inverting the container. The CMAH mutant and wild-type piglets were inoculated with 10^3^ TCID_50_/10 mL PEDV orally by hand using a syringe.

#### Clinical observations

After inoculation with PEDV, the piglets’ behaviour, including vomiting, diarrhoea, and lethargy, was observed and recorded every 4 hours for 3 days. When the piglets died or at the end of the experiment, their body weights were recorded, and they were necropsied on the same day.

#### Sampling

The intestines of all piglets were sampled at the upper and middle region of the jejunum and the upper part of the ileum by resecting a portion of the intestine approximately 10 cm in length. This piece was then ligated at both ends with surgical string, cut down, and a suitable amount of 10% formalin was injected into the luminal space. The entire sample was then immersed in ~15 mL 10% formalin and fixed for at least for 24 hours.

### Hematoxylin eosin (H/E) and immunofluorescence (IF) staining

After fixation, the samples of intestine obtained from the piglets were sliced, embedded in paraffin, and sectioned at 3 to 4 μm thickness. The sections were placed on slides, de-waxed in xylene and sequentially treated with 100%, 95%, 80% and 70% ethanol; the slides were then stained by H/E. For IF staining, the slides were de-waxed in xylene and 100% ethanol and further heated in boiling TAE buffer for 3 min to activate the antigen. After cooling to room temperature, the slides were washed with PBS for 15 min, and the tissues were stained with a primary antibody against PEDV (prepared by Dr. CM Chen) and a commercial secondary antibody, FITC-conjugated goat anti-mouse immunoglobulin (Cappel). After immersion in DAPI solution, the slides were sealed with 10% glycerol, and the signals were observed on an Olympus BX50 microscope (Olympus, Japan) enlighten by UV-light.

### Pathology evaluation

**Fig 2:**
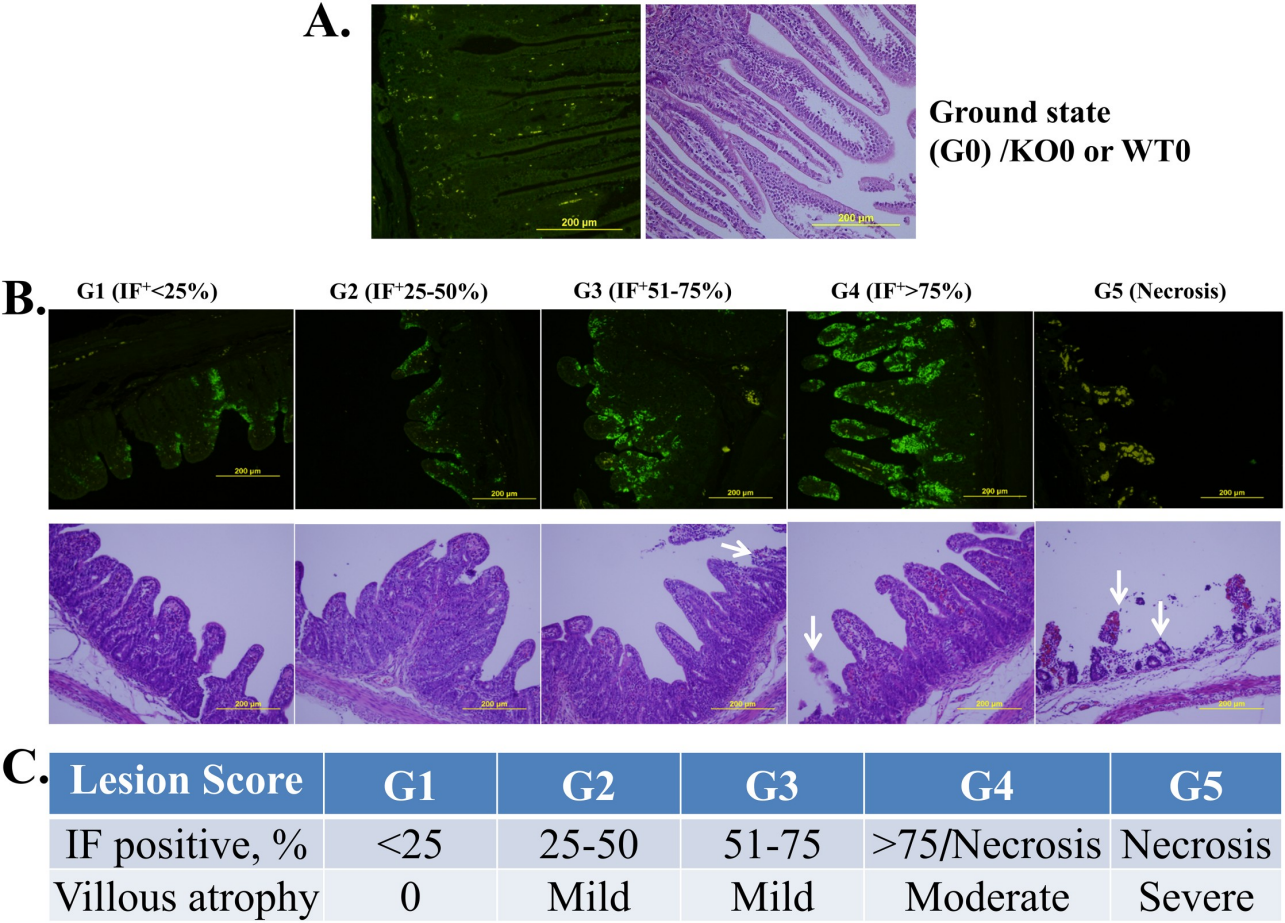
Evaluation criteria based on immunofluorescence staining and histopathological lesions (I/H score) of piglets’ intestine samples after PEDV challenge. A. KO0 or WT0 controls for the ground state, G0. B. IF is scored as G1 to G4 based on the relative intensity of staining, whereas G5 is based on villar atrophy or defoliation observed by H/E inspection. The corresponding scores are shown in C. The arrows indicate necrotic villi; the yellow bars represent 200 μm.

The criteria used to score immunofluorescence (IF) staining and histopathological lesions (I/H score) associated with PEDV are shown in Fig 2. PEDV mainly infects the epithelial cells that form the mucosa of the small intestine. Each part of the small intestine was evaluated at three different locations and their mean represented one piglet data. In the early stage of PEDV infection, only IF staining allows us to observe whether or not epithelial cells have been infected by PEDV. Therefore, at that stage, the percentage of IF-positive cells was the only criterion used to determine the severity of PEDV infection. However, in the middle to late stages of infection, the severity of PEDV infection is better judged by the degree of villar atrophy because infected cells often defoliate from the mucosa and IF may not reveal the PEDV-infected cells. Therefore, the lesions were scored from 1 to 5 as shown in Fig 2C; the scores combined the results of both IF staining and histopathological inspection in an I/H score that was used in the final statistical analysis.

### Statistical analysis

All of the clinical and viability data were recorded and analysed using GraphPad Prism 6 (GraphPad Software, Inc.). The survival rate (curves) of the piglets after PEDV challenge was analysed using the Log-rank (Mantel-Cox) and Gehan-Breslow-Wilcoxon tests. The t-test was used to analyse the body weight and immune/histopathologic data obtained from the intestinal samples from all experimental piglets. The significance level (*) was set at 0.05.

## Results

### Generation of CMAH mutant pigs

**Fig 3:**
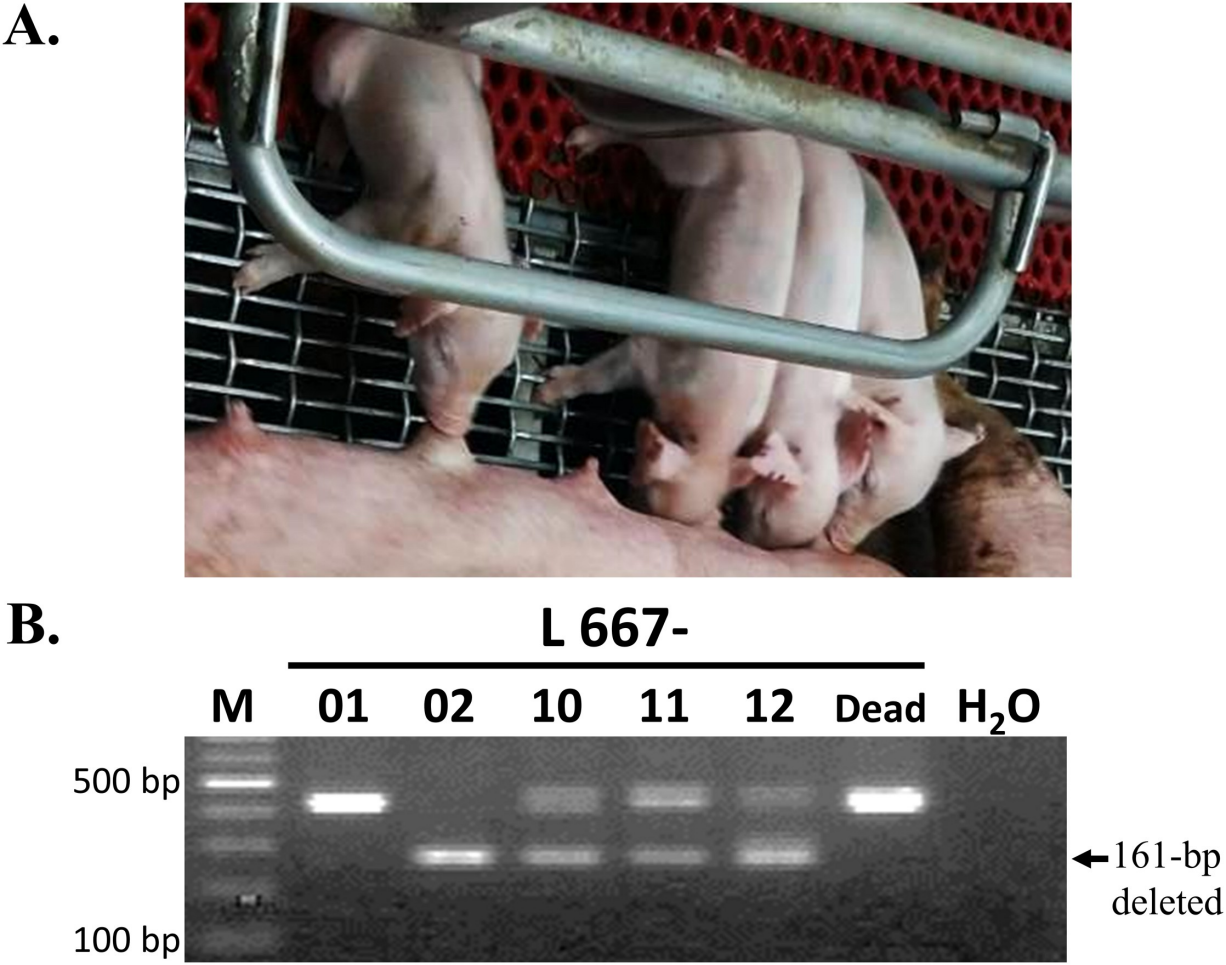
Generation of CMAH gene-edited pigs. A. Four lines of CMAH gene-edited piglets (1 male, L667-02, and 3 females, L667-10, 11, and 12) were obtained. B. CMAH KO piglets were analysed and screened by PCR. The amplicons were produced a 161-pb deleted band when two sites editing occurred simultaneously.

**Fig 4:**
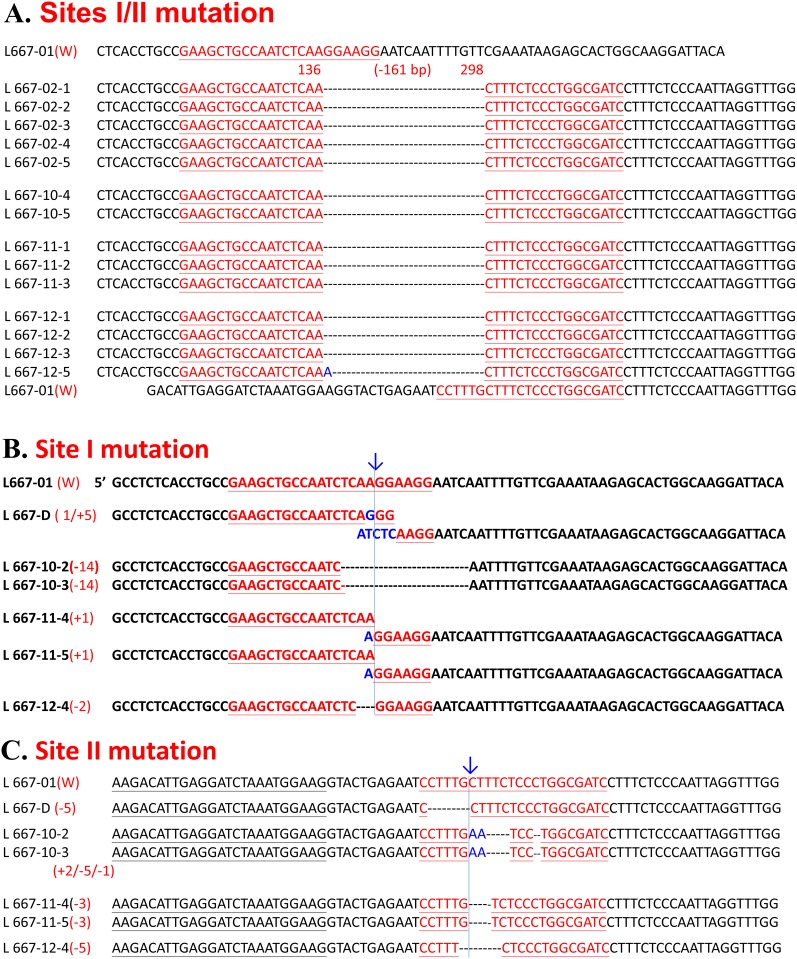
Genotyping by TA-cloning and sequencing of the porcine CMAH gene edited by CRISPR/Cas9 vectors directed against two sites. A. The genotype shown displays two simultaneously mutated sites and a deleted 161-bp DNA fragment; the blue A represents an extra inserted base that appeared in L667-12. B. The indel occurred at site I of exon II of the CMAH gene. C. Details of the mutation at site II of intron 2 of the CMAH gene. The blue arrows and lines indicate the cutting site of Cas9. The blue letters represent inserted bases, and the dashed line indicates deleted bases.

A total of 70 zygotes (Table 2) were microinjected with the CRISPR RNA, including two sgRNA, which are directed against two sites on CMAH within exon2 and intron 2 (Fig 1), and Cas9 mRNA and transferred to 3 foster dams. Five live piglets and 1 stillborn piglet were delivered by one pregnant sow (Table 2). PCR analysis of CMAH KO revealed that 1 male (L667-02) and 3 females (L667-10, -11, and -12) (Fig 3A) carried 161-bp deletion mutations (Fig 3B). Further analysis by PCR-directive sequencing (PDS) and subcloning of PCR products in T-A cloning vectors and sequencing (PTS) showed that the 4 live piglets and the stillborn piglet were biallelic CMAH mutants; of these, L667-02 was biallelic 161-bp deleted (D/D type) (Fig 4A), L667-10, -11 and -12 were mosaic with D/D type and 2 sites mutated (D/D and D/M types) (Fig 4A–4C), and the stillborn animal (L667-D) had a single base mutation and a 5-bp insertion at site I and a 5-bp deletion at site II (M/M type) (Fig 4B and 4C). The efficiency of gene editing and KO was 7.5% based on the number of manipulated embryos and 83.3% based on the number of delivered piglets, all of which were biallelic mutants (Table 2).

**Table 2:** Generation of CMAH knockout (KO) pigs by direct microinjection of sgRNA/Cas 9 mRNA into the cytoplasm of pronuclear newly fertilized porcine eggs.

Micromanipulation		No. of surrogate		No. of piglets		
Lot	No. of zygotes	Dam	Pregnant (%)	Born^a^	KO (%)	BKO^b^(%)
1	31	1	1	5/1	5 (83.3)	5 (83.3)
2	21	1	0	0/0	0 (0)	0 (0)
2	18	1	0	0/0	0 (0)	0 (0)
Total	70	3	1 (33.3)	5/1	5 (83.3)	5 (83.3)

^a^. alive/dead

^b^. biallelic knockout.

**Fig 5:**
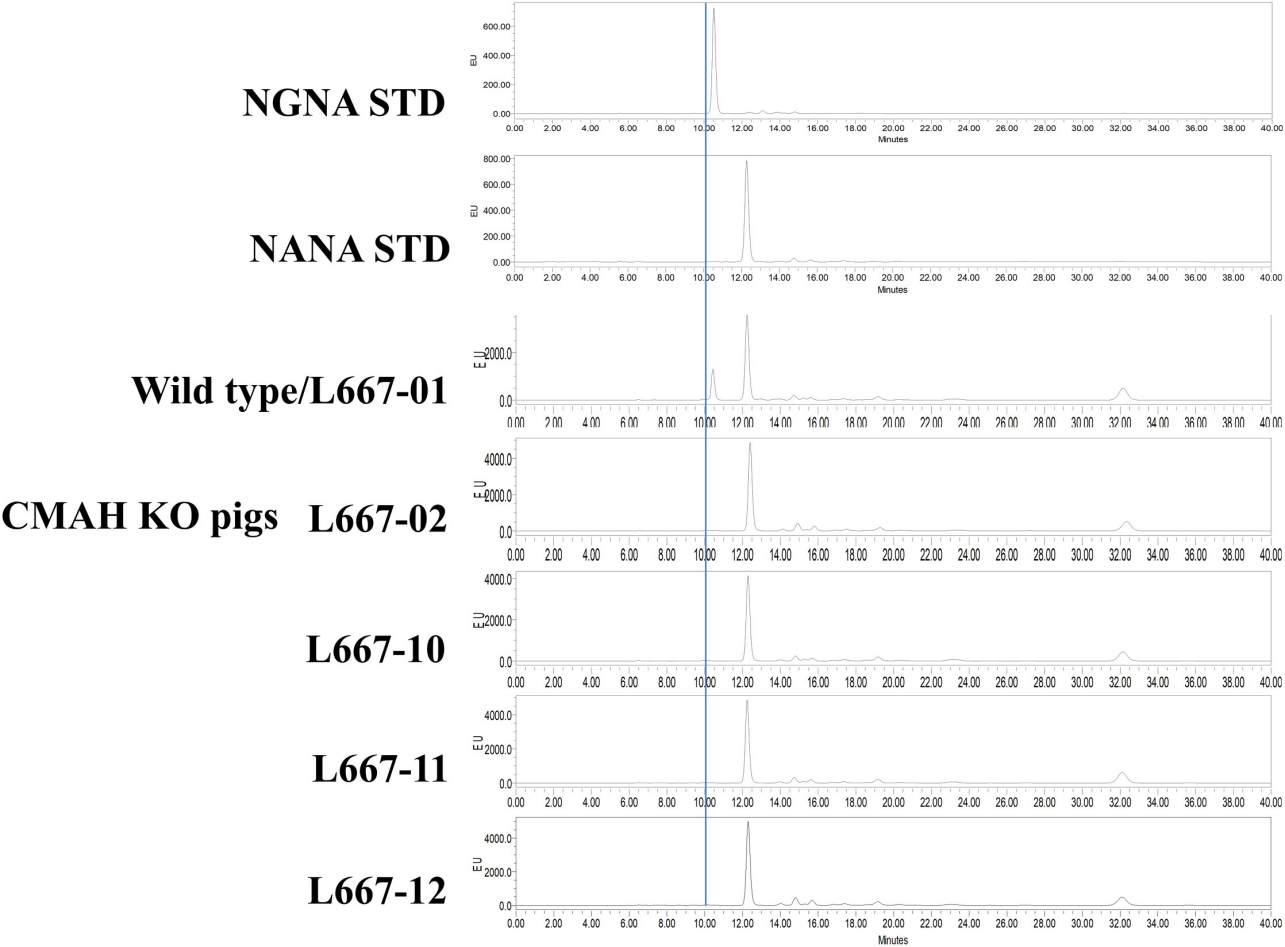
Expression of NGNA/NANA in the tissues of CRISPR/Cas9 CMAH mutant founders. L667-02, -10, -11 and -12 and their wild-type littermate (L667-01) were analysed by HPLC. NGNA STD and NANA STD are standard samples of NGNA and NANA, respectively. The blue line indicates a retention time of 10 min.

The animals used for PEDV challenge were obtained by breeding the three founders (L667-10, 11 and 12) with the founder boar (L667-02). The mutational status of their offspring (Table 3) was confirmed by PCR, PDS and PTS (S2–S4 Figs). All piglets were rapidly screened by PCR, and D/D piglets were preferentially used in the experiments. In exps. II and III, the D/D piglets were supplemented with 1 and 3 D/M type piglets, respectively (Table 4) that were confirmed by PTS to have 1-bp insertions or 14- or 2-bp deletions at site I on the mutated chromosome (S2–S4 Figs). The null expression of the CMAH gene was analysed based on the detection of NGNA/NANA by HPLC; the results showed that all founders (Fig 5) and their offspring (S5 Fig) lacked NGNA expression. These results show that all founders and their offspring are biallelic mutants that fail to express CMAH and produce no NGNA in their tissues.

**Table 3:** Germline transmission and genotypes of F1 CMAH KO piglets.

Sow	Parity	Litter size	m/f/d(n)^a^	Birth weight (Mean±SE), kg	No. of piglets of KO genotype^b^		
					D/D (-161/+1/-5)		D/M (site I)
L667-10							(-14 bp)
	1	12	5/5/2 (3)	1.43 ± 0.16	6	(5/1/0)	6
	2	8	5/3/0 (0)	1.76 ± 0.08	7	(7/0/0)	1
	3	12	3/6/3 (0)	1.53± 0.06	9	(9/0/0)	3
L667-11							(+1 bp)
	1	6	4/1/1 (0)	1.77 ± 0.05	4	(4/0/0)	2
	2	6	4/2/0 (0)	1.76 ± 0.10	3	(3/0/0)	3
	3	1	0/0/1 (0)	1.72	1	(1/0/0)	0
L667-12							(-2 bp)
	1	13	7/3/3 (3)	1.46 ± 0.10	13	(7/5/1)	0
	2	13	8/4/1 (0)	1.57 ± 0.08	11	(6/3/2)	2
	3	12	6/3/3 (0)	1.62 ± 0.07	10	(5/3/2)	2
Sum		58	33/18/7 (6)	1.58 ± 0.04	17	11/5	8

^a^. m/f/d (n): No. of males/females/stillbirths (n = live piglets that died due to weakness).

^b^. Genotypes: D indicates deleted and M refers a site I mutation in exon 2 and null CMAH expression. D/D type: -161 indicates a genomic type featuring a 161-bp deletion in which the mutation at sites I and II occurs simultaneously on both chromosomes; +1 indicates the indel with a 161-bp deletion and a 1-bp insertion (+1); and -5 indicates the presence of a 161-bp deletion with simultaneous deletion of 5 additional bp (-5 bp). D/M type: -161 bp/ site I mutated; in parentheses, -14 bp indicates a 14-bp deletion, +1 bp indicates a 1-bp insertion, and -2 bp indicates a 2-bp deletion.

**Table 4:** Genotypes of the F1 CMAH KO piglets used for PEDV challenge.

Exp.	Sow/ L667-			Genotype		Site I of Mutant allele
	Parity	ID.	Litter size	D/D	D/M	
1	1	10	12	1	0	-
		11	6	2	0	-
		12	13	3	0	-
Control		12		1	0	-
2	2	10	8	3	0	-
		11	6	3	1	+1
		12	13	2	0	-
Control		12		1	0	-
3	3	10	12	4	2	-14
		11	1	0	0	-
		12	12	5	1	-2
Control		10		1	0	-

Note: Genotypes: D indicates that the mutation occurred at two sites simultaneously and resulted in a 161-bp deletion, whereas M is site I-mutated, on codon region and null CMAH expression; D/D refers to the case in which the 161-bp deletion occurred on both chromosomes. The controls are wild-type piglets.

### Clinical observation of neonatal piglets challenged with nv-PEDV

**Fig 6:**
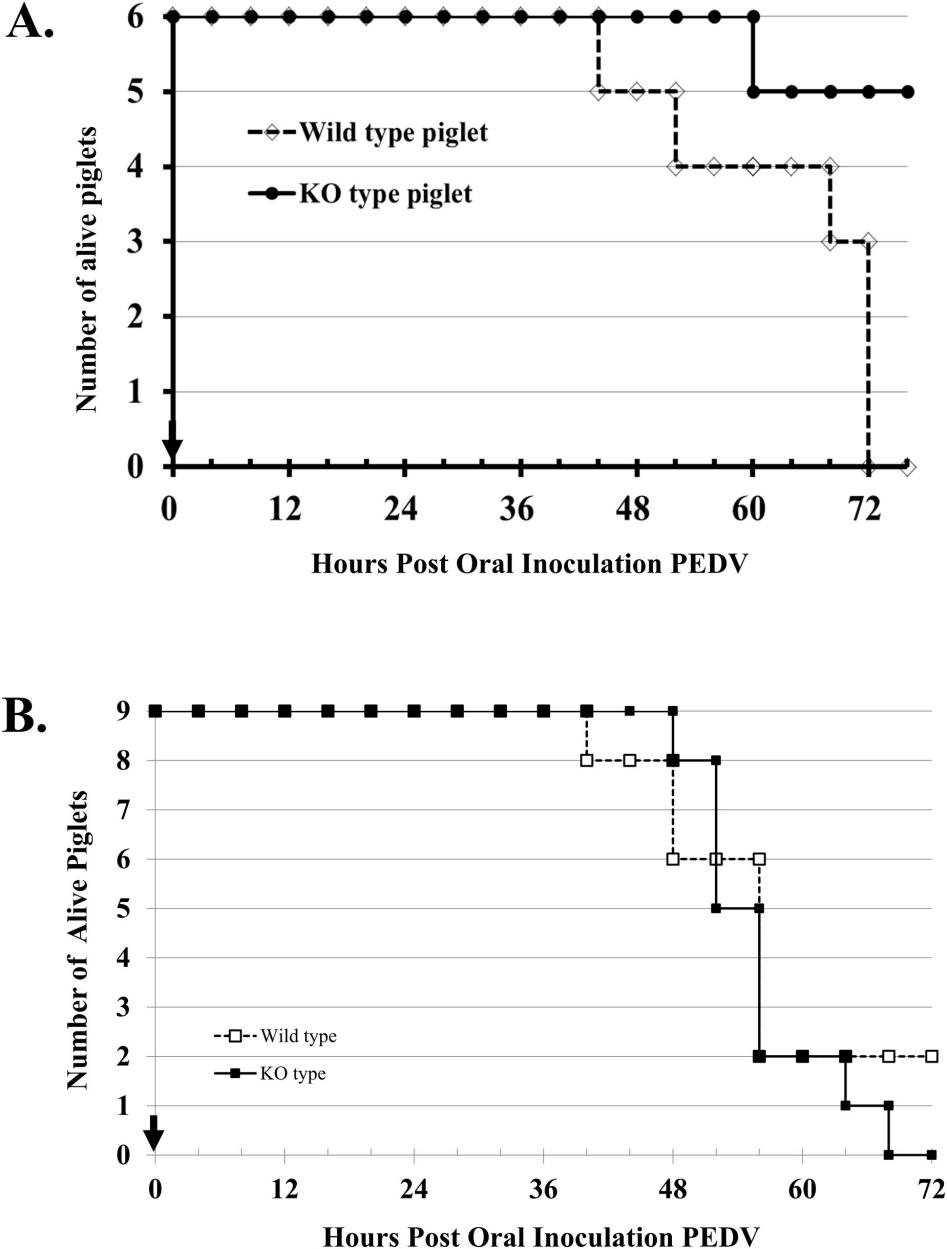
Survival of neonatal piglets after oral inoculation with nv-PEDV. A (Exp. I), 2-day-old piglets’; and B (Exp. II), 3-day-old neonatal piglets’ survival curve after inoculated with nv-PEDV. Solid circles (A) or squares (B) with lines represent the CMAH KO piglets, and open diamonds (A) or squares (B) with dashed lines indicate wild-type piglets. The arrow shows the time of inoculation. In A at 72 hpi, three moribund WT piglets are classified as dead piglets.

**Fig 7:**
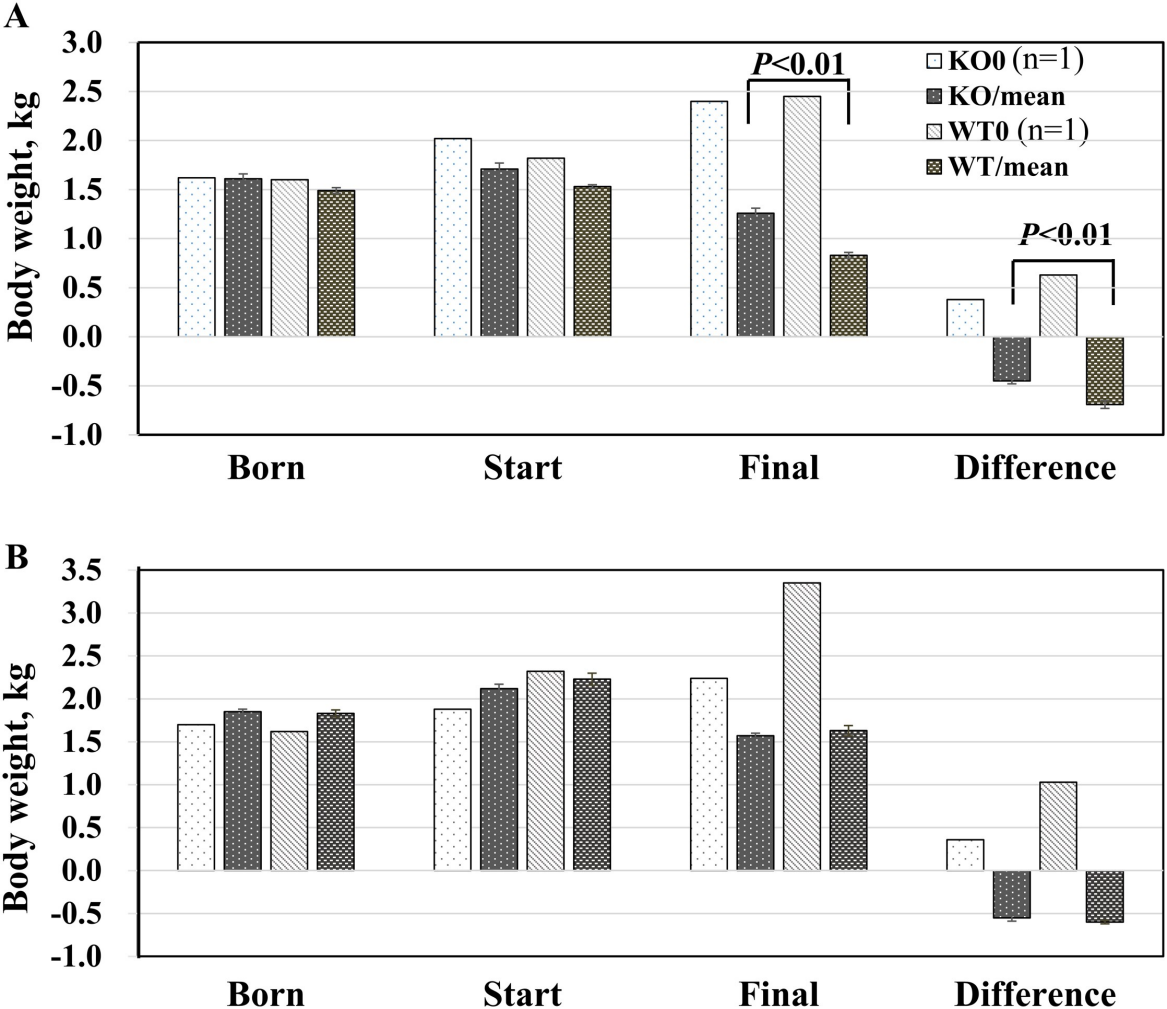
Body weights of neonatal piglets before and after oral PEDV inoculation. A. 2-day-old piglets (n = 6) and B. 3-day-old piglets (n = 9) in Exp. I and Exp. II, respectively, show body weights changes 72 h after inoculated with nv-PEDV. K0 and W0 represent KO and WT animals that were not inoculated with PEDV and were reared by their dams on the farm. KO and WT are knockout treated and wild-type treated animals, respectively.

**Exp. I**: When neonatal 2-day-old piglets were challenged with nv-PEDV, both the CMAH mutant (Knockout, KO) and wild-type (WT) animals initially displayed clinical signs of vomiting and diarrhoea at 12 hours post-inoculation (hpi), and their activity also decreased (Table 5). In the WT group, the first piglet’s death occurred at 44 hpi; a second animal died at 52 hpi, a third at 68 hpi, and the remaining three animals were moribund and nearly dead at 72 hpi (Fig 6A). In the CMAH KO group, the first piglet died at 60 hpi, 3 piglets were moribund at 72 hpi, and the other remaining two piglets survived until the end of the trial (Fig 6A and Table 5). After nv-PEDV inoculation, the loss of body weight of WT piglets was 0.69±0.04 kg, significantly (p<0.01) greater than that of CMAH KO piglets (0.45±0.03 kg) (Fig 7A).

**Table 5:** Clinical signs displayed by neonatal piglets after nv-PEDV inoculation in Exps. I and II.

Exp.	Geno-type	No.	Hours post nv-PEDV inoculation						
			4–8	12	24	36	48	60	72
I	KO	6	6A/	6B/	6B/	1A5B/	6B/	4B1C1D/	2B3C1D/
			6n	2d4dv	3n3d	4n2d	3n3d	1n4d	5d
	WT	6	6A/	6B/	2B4C/	5B1C/	4B1C1D/	1B3C2D/	3C3D/
			6n	6dv	2n4d	6d	2n2d1dv	4d	3d
II	KO	9	9A/	9A/	9B/	9B/	6B2C1D/	1B1C7D	9D/
			9n	2n1v2d4dv	2n6d1dv	9d	8d	2d	0
	WT	9	9A/	9A/	9B/	9B/	5B2C2D/	2B7D/	1B1C7D/
			9n	4n3v1d1dv	1n8d	1n8d	7d	2d	2d

Note: No. of piglets with viability and clinical signs: viability—A is normal, B indicates decreased activity, C is moribund, and D indicates dead; clinical signs—n indicates no clinical signs, d is diarrhoea, and v is vomiting.

**Exp. II**: The 3-day-old piglets were examined as in exp. I. Although both CMAH KO and WT animals initially showed clinical signs of vomiting and diarrhoea at 12 hpi, 2 KO and 4 WT piglets were without clinical signs (Table 5). Furthermore, all piglets sustained their activity until 24 hpi (Table 5). In the WT group, the first death occurred at 40 hpi (Fig 6B); two piglets were lost at 48 hpi, 4 piglets died at 56 hpi, and the remaining two piglets were alive at the end of the trial. In the CMAH KO group, the first animal was lost at 44 hpi, and 3, 3, 1 and 1 piglets died at 52, 56, 64 and 68 hpi, respectively (Fig 6B). There was no significant difference in the decrease in body weight in the two groups of piglets (WT/ -0.60±0.02 kg vs. CMAH KO/-0.55 ±0.04 kg; p> 0.05) (Fig 7B).

**Exp. III**: To examine the early events and the role of NGNA in nv-PEDV infection of neonatal piglets, we used 2-day-old piglets challenged with nv-PEDV. After infection, the piglets were fed sows’ milk and skim milk every 4 hours for 24 hours; this was then replaced by Ringer’s lactate solution supplemented with 5% glucose, and the piglets were sacrificed at 24, 48 and 72 hpi. The results (Table 6) show that until 12 hpi both the CMAH KO and WT piglets appeared normally active; however, with respect to clinical signs, only 3/11 CMAH KO piglets did not show diarrhoea or vomiting at 12 hpi. From 4 to 24 hpi, all piglets were fed their own dams’ whole or skim milk; the results show that all piglets displayed decreased activity and diarrhoea without a significant difference between CMAH KO and WT piglets. One moribund CMAH KO piglet was observed at 44 hpi, and one moribund WT piglet was observed at 56 hpi; all of the piglets stopped vomiting after 24 hpi. After the sow’s milk was replaced with RLG, all piglets (both CMAH KO and WT) showed sustained activity and viability at least until 56 hpi, with the exception of one CMAH KO piglet that died prior to the end of the trial (Table 6).

**Table 6:** Clinical signs displayed by neonatal piglets after nv-PEDV inoculation in Exp. III.

Geno-type	h	No.		Hours post nv-PEDV inoculation									
			4–8	12	16	20	24	28–40	44	48	56	64	72
KO	24	3	3A/	3A/	3B/	2B1C/	3B/	-	-	-	-	-	-
			3n	2n1v	3d	1v2d	3d						
	48	2^#^	2A/	2A/	2B/	2B/	2B/	2B/	1B1C/	2B/	-	-	-
			2n	1n1v	2d	1d1dv	2d	2d	1n1d	1n1d			
	72	6	6A/	6A/	6B/	6B/	6B/	6B/	6B/	6B/	4B2C/	3B3C/	3B2C1D
			6n	4v1d1dv	6d	6d	6d	6d	6d	6d	6d	6d	/5d
WT	24	3	3A/	3A/	3B/	2B/	3B/	-	-	-	-	-	-
			3n	2v1dv	3d	2d1dv	3d						
	48	3	3A/	3A/	3B/	3B/	3B/	3B/	3B/	3B/	-	-	-
			3n	1v1d1dv	3d	3d	3d	3d	3d	3d			
	72	6	6A/	6A/	6B/	6B/	6B/	6B/	6B/	6B/	5B1C/	4B2C/	5B1C/
			6n	2v4dv	6d	6d	6d	6d	1n5d	6d	6d	6d	6d

Note: No. of piglets with viability and clinical signs: viability—A is normal, B indicates decreased ability, C is moribund, and D is dead; clinical signs—n indicates no clinical signs, d is diarrhoea, and v is vomiting.

^#^ One piglet died before experiment.

### Immuno/Histopathology of neonatal piglets challenged with nv-PEDV

**Fig 8:**
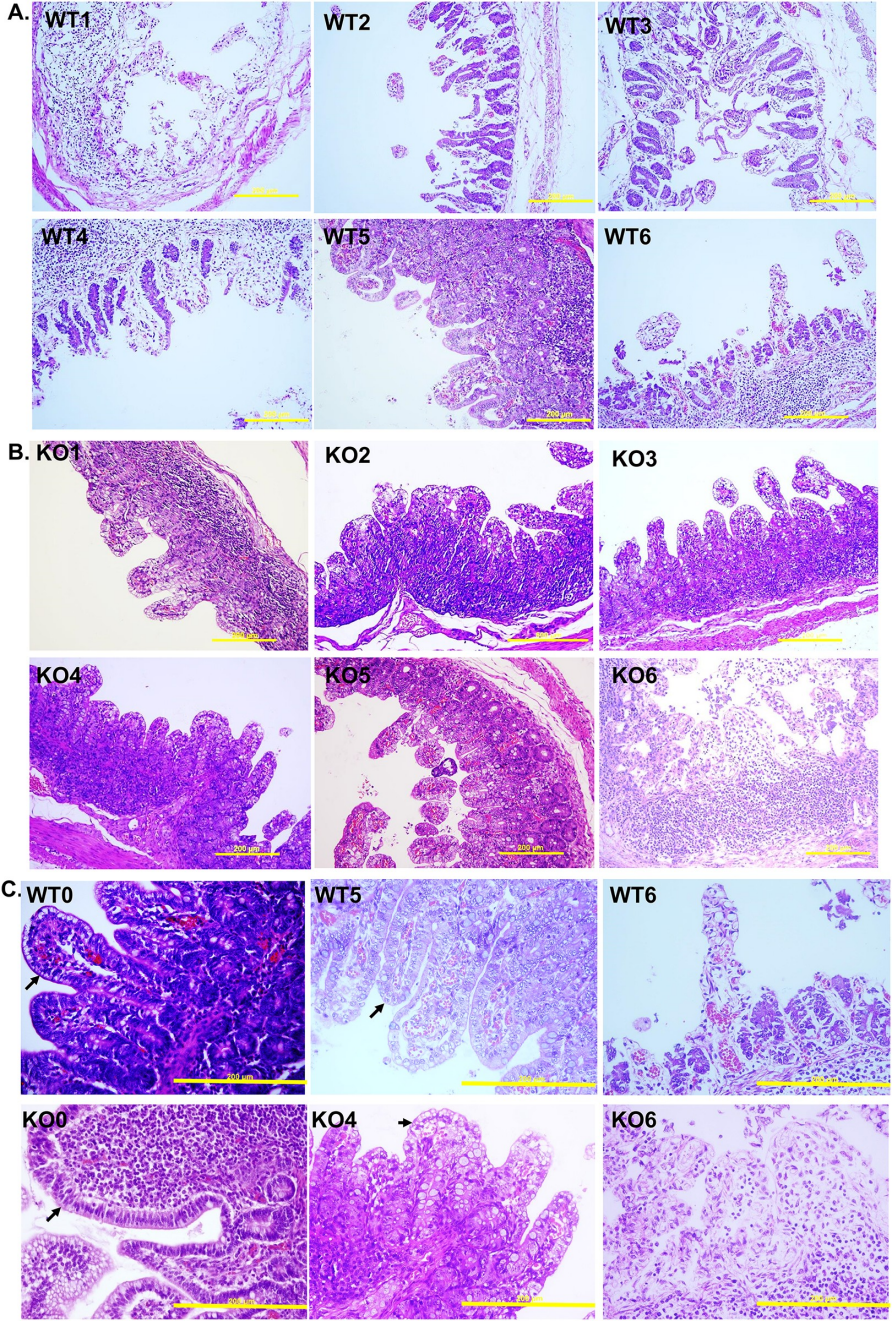
Pathological inspection of piglets’ intestine at the middle jejunum by H/E staining. Panels A. and B. indicate wild-type and knockout piglets, respectively, after PEDV oral inoculation. C. The samples from control, the best and the worst pathological responded piglets, which are no nv-PEDV-inoculated, survival and dead, respectively, at 72 hpi. The upper panels (WT0, WT5 and WT6) are samples from wild type piglets and the down panels (KO0, KO4 and KO6 are samples from CMAH KO piglets. Arrows indicate epithelial cells and the yellow bars indicate 200 μm.

**Fig 9:**
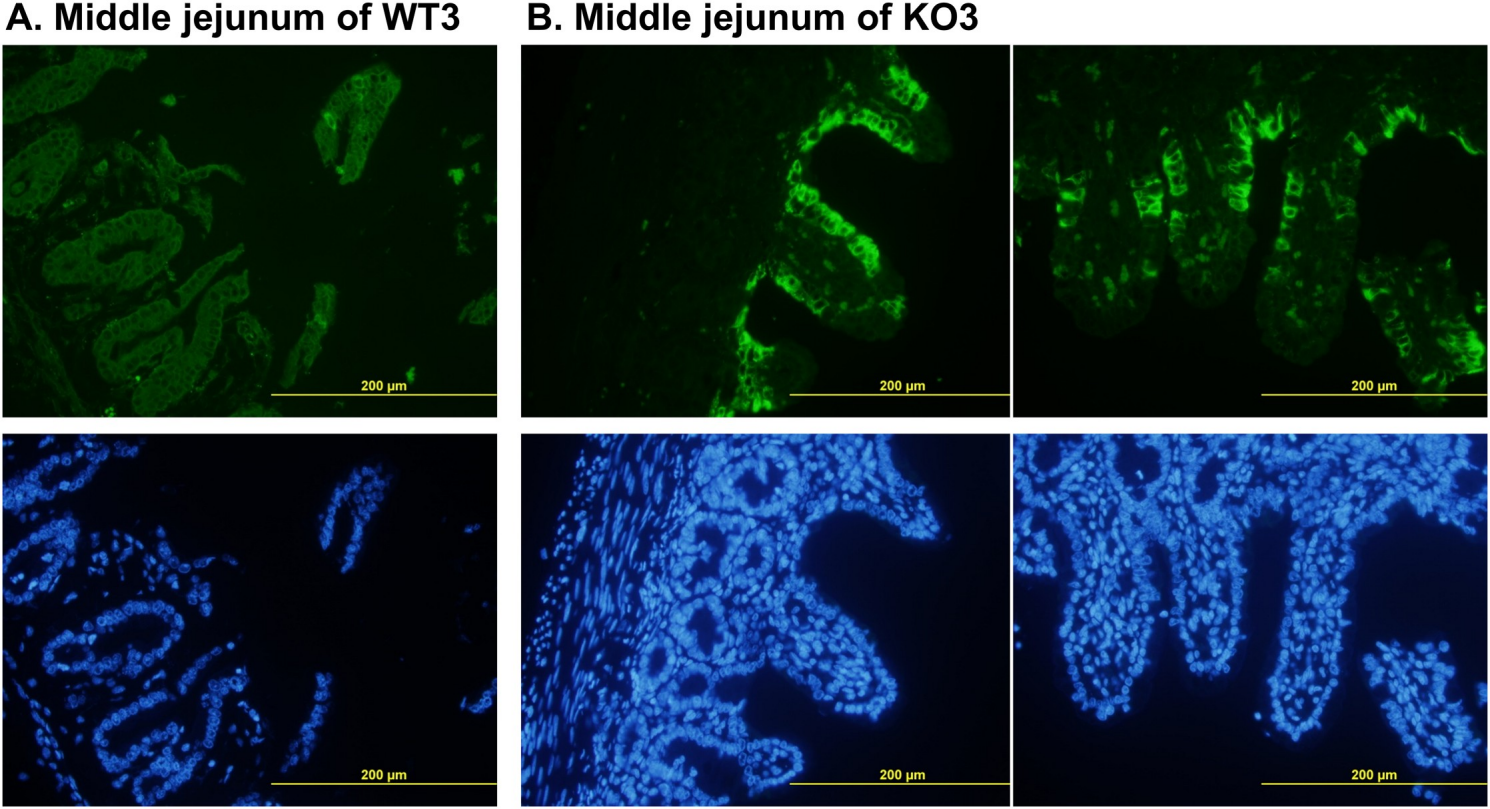
Immunofluorescence staining with an antibody against nv-PEDV N protein. WT3 (A) shows a sample from a wild-type piglet, and KO3 (B) shows a sample from a double-chromosome CMAH gene knockout piglet. The samples shown in the upper, green colour, and lower, blue colour, panels are fluorescent stained with nv-PEDV antibody and DAPI, respectively. The yellow bars indicate 200 μm.

After 72 hpi, all of the dead and euthanized piglets were necropsied, and their intestines were sampled for pathological examination. Grossly, the small intestine appeared transparent and orange-yellow to flesh pink in colour; it was thin-walled and dilated with fluid content in the live piglets (S6A and S6B Fig). In exp. I, the PEDV induced histopathologic changes, including enterocyte necrosis, degeneration, and exfoliation, and collapsed lamina proprial tissues containing karyorrhectic debris, were noted in all challenged piglets. However, these lesions varied from mild to severe, and the lesions were more severe in the moribund WT piglets than in the CMAH KO piglets (Fig 8). Immunofluorescence (IF) staining with a monoclonal antibody against PEDV nuclear protein was used to detect PEDV antigens. The results showed that PEDV antigen was presented in the epithelium covering the moderately atrophic tips of villi in the small intestine of WT and CMAH KO piglets (Fig 9). However, if the epithelial cells were defoliated from the villi after PEDV infection, no positive signals would be expected (Fig 9A). We further scored the severity of lesions in the intestines of the infected animals (Fig 2) by a combination of IF staining and histopathological inspection (immuno/histopathological, I/H, score). According to I/H scores, results revealed that the severity of the intestinal lesions in KO piglets (3.7±0.3 to 4.2±0.2) less than those in WT piglets (4.8±0.2) (*p*<0.05) in exp. I; but there were no significant difference among WT piglets (from 3.4±0.6 to 4.4±0.3) and CMAH KO piglets (from 4.3±0.4 to 4.7±0.2) in exp. II (Table 7). In exp. III, even ruling out the possible effects of feeding the animals with commercial baby cow milk, we also found no significant difference in I/H scores of WT and CMAH KO piglets (Table 8). According to the I/H scores obtained at 72 hpi, which ranged from 3.8±0.4 to 3.2±0.5 in CMAH KO piglets and from 2.8±0.4 to 2.5±0.2 in WT piglets (*p*>0.05), most piglets seemed to improve compared with those at 24 and 48 hpi when offered sow’s milk and supplemental lactated Ringer’s solution containing 5% glucose (Table 8).

**Table 7:** The immunofluorescence and histopathological (I/H) score of piglet small intestine at 72 h after oral Inoculation of 2 (I)- or 3 (II)-day old neonates with PEDV.

Exp.	Genotype	n	Jejunum		Ileum
			Front	Middle	
**I**	KO	6	4.0±0.3^b^	4.2±0.2^b^	3.7±0.3^b^
	WT	6	4.8±0.2^a^	4.8±0.2^a^	4.8±0.2^a^
**II**	KO	9	4.3±0.4	4.7±0.2	4.4±0.4
	WT	9	4.4±0.3	3.4±0.6	3.6±0.6

KO = CMAH KO homozygotes, WT = wild-type piglets.

^a, b^. In exp. I, means between the same columns differ significantly (p<0.05).

**Table 8:** Intensity of PEDV infection of epithelial cells of villi in CMAH KO piglets’ intestines revealed by immunofluorescence and histopathological (I/H) score.

hpi^1^	Genotype^2^	No. of piglets	Jejunum		Ileum.
			Front	Middle	
24	KO	3	5.0±0.0	5.0±0.0	3.3±0.9
	WT	3	3.0±1.0	4.0±0.6	3.7±0.9
48	KO	2	4.0±0.0	4.0±0.0	4.0±0.0
	WT	3	4.0±0.0	4.0±0.0	3.7±0.3
72	KO	6	3.2±0.5	3.8±0.4	3.3±0.6
	WT	6	2.5±0.2	2.8±0.4	2.5±0.3

^1^. hpi = hours post inoculation.

^2^. KO = CMAH gene knockout by gene editing; WT = wild-type piglets.

## Discussion

Currently, gene editing is widely used in both basic and applied studies, e.g., in studies of the disease resistance of farm animals [33]. One convincing report showed that CD163 gene-edited pigs generated by CRISPR/Cas9 exhibited physiological normality and showed little vulnerability to porcine reproductive and respiratory syndrome virus (PRRSV) infection either *in vitro* [34] or in vivo [35–37]. However, other attempts, including putative receptors of CD169 KO and CD163 KO, failed to produce evident resistance to PRRSV [38] or African swine fever [39], respectively. These failures may have occurred because the mechanism of viral infection involves other receptors or because it does not involve receptors [38].

The hypothesis that the absence of NGNA expression in CMAH KO piglets disables PEDV infection was partially proven in this study. In exp. I, 2-day-old old piglets were orally inoculated with the local outbreak strain nv-PEDV [31]. Although the final (72 hpi) survival rate differed little in the WT and KO animals, based on the histopathologic examination and considering the 3 deadly moribund WT piglets, the CMAH KO piglets showed greater resistance to nv-PEDV infection than the WT animals. This assumption is supported by the high degree of histopathologic severity found in the WT piglets, which clearly differed from that observed in the CMAH KO piglets. However, when 3-day-old piglets were used, no differences between CMAH KO and WT piglets were observed. It is doubtful that the NGNA present in cow’s milk-based formula would enable the virus to infect the CMAH KO piglets. In exp. III, colostrum from the KO or WT sows was given to avoid any possible NGNA inference, yet the final susceptibilities of the two genotypes were similar, due to no or little NGNA (S7 Fig). However, at least 3 of the 11 CMAH KO piglets showed normal activity and no clinical signs (no vomiting or diarrhoea) at 12 hpi, whereas the WT piglets displayed vomiting and/or diarrhoea. Lessened severity was therefore observed.

Considering that transmissible gastroenteritis virus (TGEV) and other coronaviruses use sialic acid (neuraminic acid, NA) and APN as their first [12,15] and second receptors [13,15], PEDV might act in a similar manner. Recently, the domain VII of APN was suggested to play a critical role for PEDV binding [40]; yet, when the ANPEP (APN) was null mutated by using CRISPR/Cas9 editing, the KO piglets though not infected by TGEV but still vulnerable to PEDV [41]. We found the major components of porcine mucin in the small intestinal submucosa are two types of NA, N-acetylneuraminic acid (NANA) and N-glycolylneuraminic acid (NGNA) (unpublished data). Using a glycan screening array, Liu et al. [42] showed that Neu5Ac (or NANA) has the highest binding affinity for PEDV S1-NTD-CTD; however, they also found that porcine mucin or bovine mucin could inhibit or block *in vitro* PEDV and TGEV infection of PK-15 or Huh-7 cells which transfected with porcine APN. The present results show that CMAH KO piglets exhibited delayed infection and minor symptoms after oral PEDV inoculation, suggesting that in CMAH KO piglets that are normally nursed, PEDV may be unable to bind efficiently to the APN on the villi of epithelial cells and pass through the intestinal lumen. The infection of virus via receptor could be the sole route for PRRSV [35–37] and TGEV [12,15,41], but not true for PEDV, might be through a more complicate mechanism other than receptors [43].

It is known that PEDV causes severe enteric disease in suckling piglets [44,45] and less severe disease in older weaned pigs [46]. Our results suggest that the differentiation might occur as early as in the neonatal period; clinical diarrhoea and/or vomiting and decreased activity were observed in all 2-day-old piglets but improved in 3-day-old piglets. When caesarean-delivered and colostrum-deprived (CDCD) animals were used for oral inoculation of PEDV, the 1-day-old piglets showed clinical signs at 12 hpi [47]; this was also observed in our study using naturally delivered piglets. Furthermore, in PEDV inoculation studies, 5-day-old CDCD piglets were more sensitive than 21-day-old weaned piglets [32]. Similarly, naturally delivered 9-day-old suckling piglets showed a weaker innate immune response to PEDV than weaned pigs [48]. This study used 2- or 3-day-old piglets that were naturally delivered and nursed with colostrum by CMAH KO or WT sows prior to PEDV oral inoculation in an attempt to realize the protective effects of nursing in animals in which the biallelic CMAH genes were mutated. In exp. III, the clinical symptoms of 2-day-old piglets that were PEDV inoculated and hand fed whole or skim sow’s milk for an additional 24 h were similar to those of the 3-day-old piglets in exp. II. Furthermore, when lactated Ringer’s solution supplemented with 5% glucose was offered from 24 to 72 hpi, the epithelial cells of the villi showed less damage and/or showed increased recovery of epithelial cells from the crypts according to the I/H scores, which ranged from 4.0 ± 0.0 to 2.5 ± 0.2 in WT piglets and from 5.0 ± 0.0 to 3.2 ± 0.5 in the KO group. This benefit of oral rehydration therapy in acute viral diarrhoea could be attributed to glucose-facilitated sodium absorption [49] and to alleviate Na^+^-K^+^-ATPase and Ca^2+^-Mg^2+^-ATPase damage [50]. Currently, the model may be improved by inoculating the piglets and allowed them to be continually nursed by dams of the same genotype to avoid NGNA interference.

In addition to their disease resistance, CMAH and GGTA1 KO animals are likely to display reduced hyperacute rejection of xenografts [51]. Our unpublished data also revealed that the acellular extracellular matrix derived from the intestine of CMAH KO pigs caused significantly less inflammation than that obtained from WT pigs after intramuscular implantation into CMAH/GGAT1 double KO pigs. Furthermore, NGNA present in red meat has been suggested to be a risk factor for human colorectal cancer and atherosclerosis in persons who habitually consume red meat [52]. Therefore, CMAH mutant pigs generated by GE can be viewed as pigs that offer a source of healthy red meat and of material that is suitable for use in biomedical devices.

In conclusion, the CMAH mutant pigs generated by gene editing could be a new breed with less susceptibility to PEDV, a source animal for medical materials and xenografts, and a source of healthy red meat.

## Supporting information

S1 Fig Representative map of the pT7-Flag_2_-NLS_1_-Cas9-NLS_2_-3’pA and pSP6-CMAH-sgRNA vectors. T7 and SP6 promotors are used for *in vitro* transcription Cas9 mRNA and CMAH-sgRNA, respectively. NLS1 and NLS2 are nuclear localization sequences [30]. *Eco*RI, *Mlu*I, *Acc*65I, *Hin*dIII, *Bam*HI and *Bgl*II are restriction enzyme sites. Amp is ampicillin resistance gene for plasmid selection during vectors constructing. Flag tag was used for assess Cas9 protein expression.
(TIF)

S2 Fig Analysis of CMAH gene-edited offspring from the first parity. A. The PCR products that revealed more than one band were further subcloned into the TA vector for colony purification and sequencing. B. Offspring with mutations at site I (exon II) and site II (intron 2). C. The offspring carrying two sites mutated simultaneously, with deletion of a 161-bp DNA fragment, and some of them showed further indel of +1 or -5 bp. D1 –D5 are stillborn piglets. In PCR, + and − are reaction positive and negative control.
(TIF)

S3 Fig Analysis of CMAH gene-edited offspring from the second parity. A. The PCR products that revealed more than one band were further subcloned into the TA vector for colony purification and sequencing. B. Offspring with mutations at site I (exon II) and site II (intron 2). C. The offspring carrying two sites mutated simultaneously, with deletion of a 161-bp DNA fragment, and some of them showed further indel of +1 or -5 bp.
(TIF)

S4 Fig Analysis of CMAH gene-edited offspring from the third parity. A. The PCR products that revealed more than one band were further subcloned into the TA vector for colony purification and sequencing. B. Offspring with mutations at site I (exon II) and site II (intron 2). C. The offspring carrying two sites mutated simultaneously, with deletion of a 161-bp DNA fragment, and some of them showed further indel of +1 or -5 bp.
(TIF)

S5 Fig HPLC analysis of NGNA/NANA in the ear tissues of six F1 offspring of the CMAH KO founders. **T**he retention times of NGNA and NANA are shown as numbers on the peaks.
(TIF)

S6 Fig Gross appearance of the small intestine of neonatal piglets at 72 hpi or at the time of death during the trial. **A**. The KO0 and WT0 are control piglets without nv-PEDV inoculated. B. KO1 to KO5 are survival KO piglets.
(TIF)

S7 Fig Analysis of NGNA by HPLC on colostrum from KO and WT sows or commercial baby formula. The blue line show a non-specific peak with retention time (RT) at 9.51–9.52 min and appeared in all samples. The RT of NGNA peak are 9.671–9.737 min near the non-specific peak. STD means standard samples of NGNA or NANA.
(TIF)
