# Supplementary figures and images for: Lessening of porcine epidemic diarrhoea virus susceptibility in piglets after editing of the CMP-N-glycolylneuraminic acid hydroxylase gene with CRISPR/Cas9 to nullify N-glycolylneuraminic acid expression
Source: PLoS One. 2019 May 29;14(5):e0217236. doi: 10.1371/journal.pone.0217236 (PMC6541307; doi:10.1371/journal.pone.0217236)

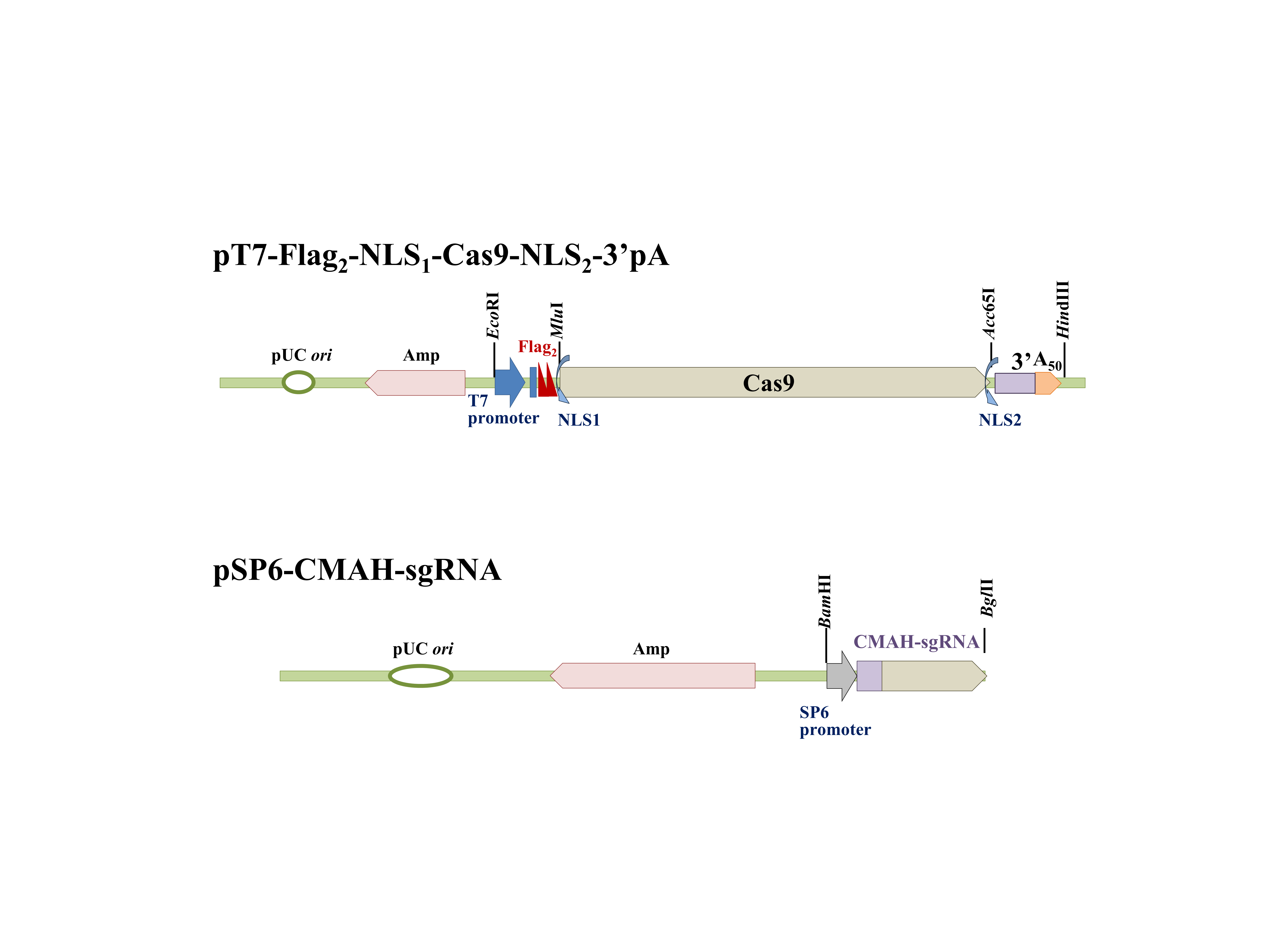

Supplement: S1 Fig — T7 and SP6 promotors are used for in vitro transcription Cas9 mRNA and CMAH-sgRNA, respectively. NLS1 and NLS2 are nuclear localization sequences [30]. EcoRI, MluI, Acc65I, HindIII, BamHI and BglII are restriction enzyme sites. Amp is ampicillin resistance gene for plasmid selection during vectors constructing. Flag tag was used for assess Cas9 protein expression. (TIF) [file pone.0217236.s001.tif]

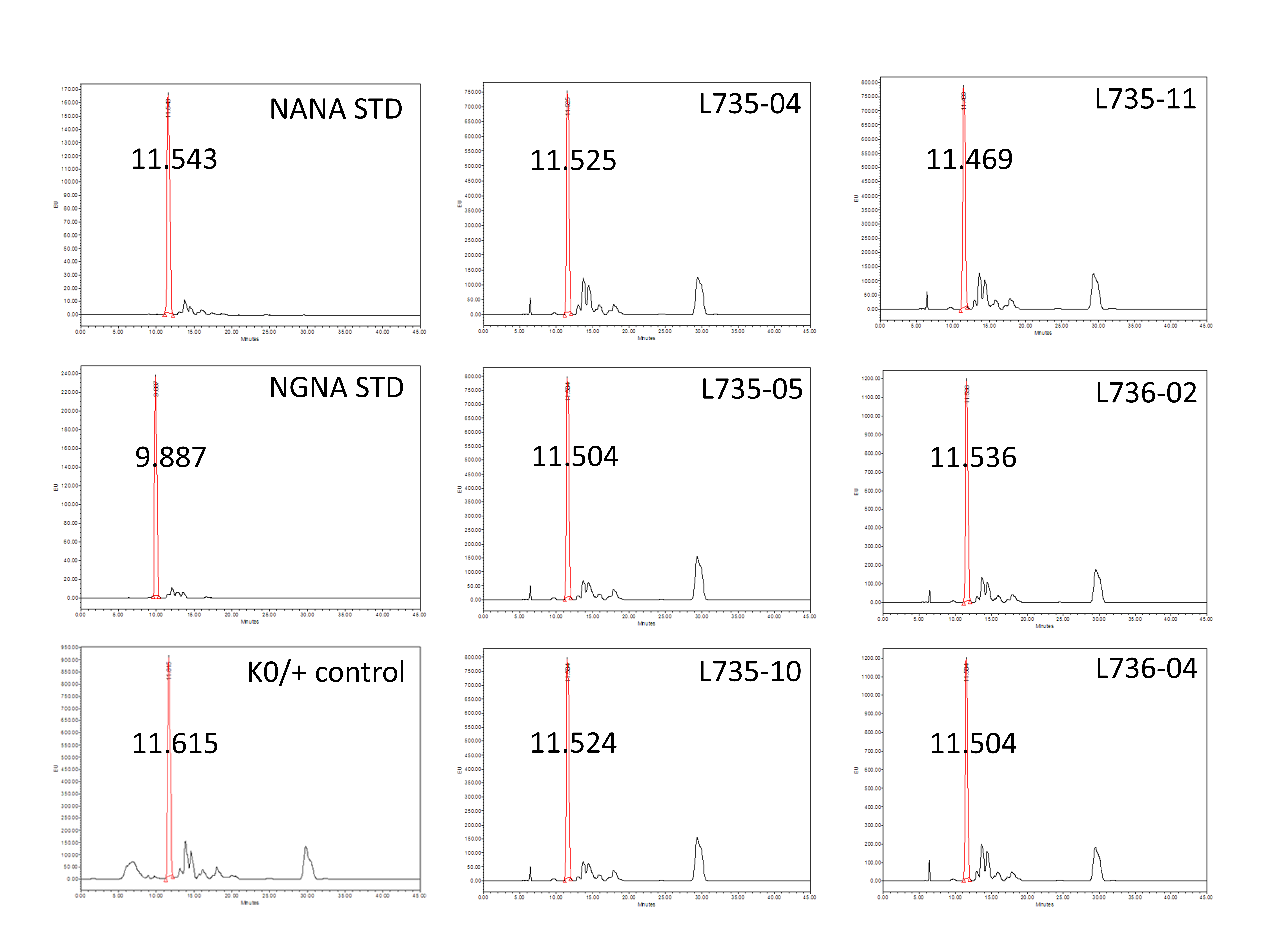

Supplement: S5 Fig — The retention times of NGNA and NANA are shown as numbers on the peaks. (TIF) [file pone.0217236.s005.tif]

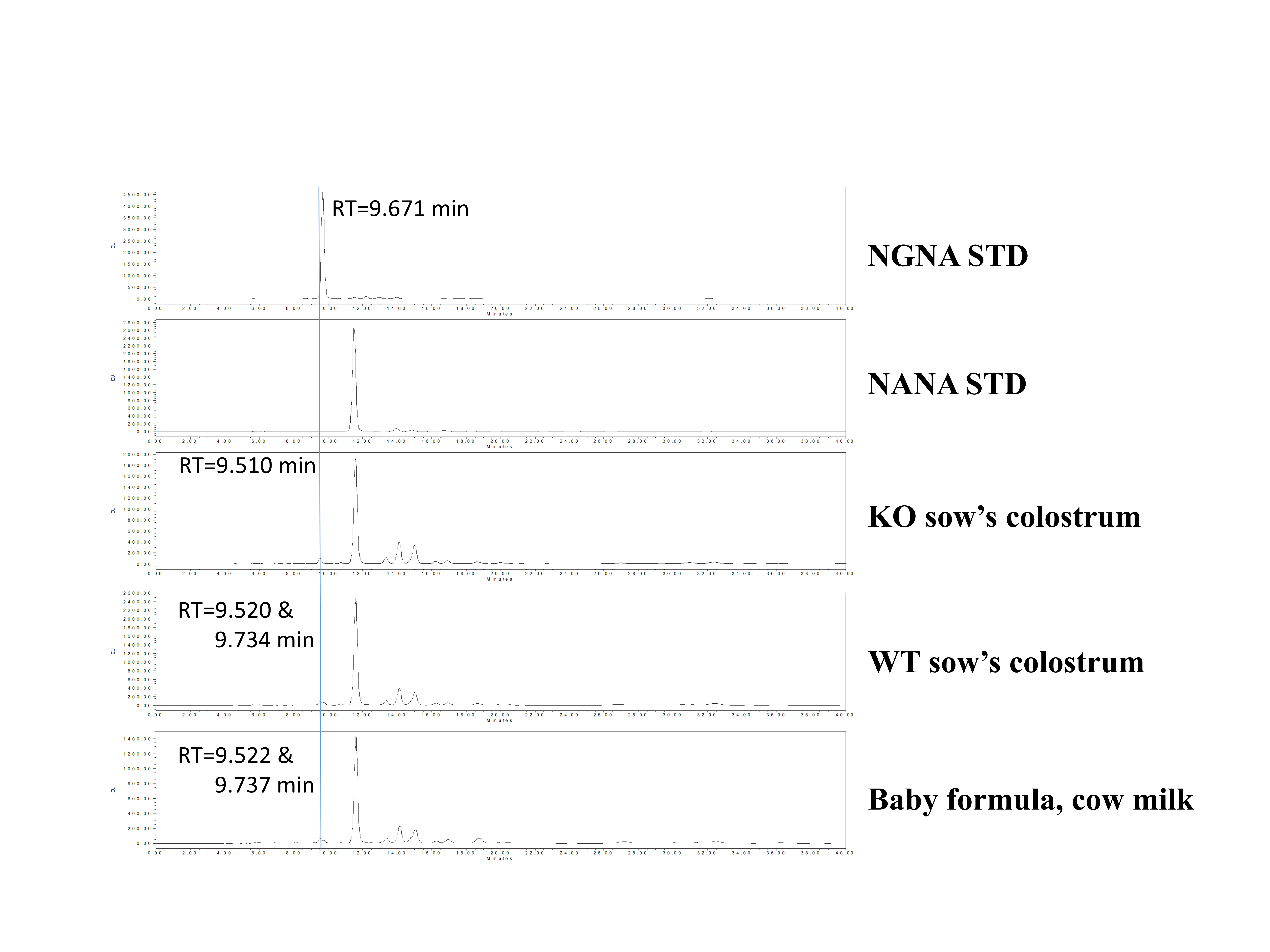

Supplement: S7 Fig — The blue line show a non-specific peak with retention time (RT) at 9.51–9.52 min and appeared in all samples. The RT of NGNA peak are 9.671–9.737 min near the non-specific peak. STD means standard samples of NGNA or NANA. (TIF) [file pone.0217236.s007.tif]
